# Predicting others’ actions from their social contexts

**DOI:** 10.1038/s41598-023-49081-6

**Published:** 2023-12-12

**Authors:** Shaheed Azaad, Natalie Sebanz

**Affiliations:** Central European University, Vienna, Austria

**Keywords:** Psychology, Human behaviour

## Abstract

Contextual cues have been shown to inform our understanding and predictions of others’ actions. In this study, we tested whether observers’ predictions about unfolding actions depend upon the social context in which they occur. Across five experiments, we showed participants videos of an actor walking toward a piece of furniture either with (joint context) or without (solo context) a partner standing by it. We found greater predictive bias, indicative of stronger action expectations when videos contained a second actor (Experiment 1), even when the solo condition had a perceptually-matched control object in place of the actor (Experiment 2). Critically, belief manipulations about the actions the walking actor would perform suppressed the difference between social context conditions when the manipulation specified an action possible in both contexts (Experiment 5) but not when the action was one that would be difficult without a partner (Experiment 4). Interestingly, the social context effect persisted when the belief manipulation specified an unlikely action given the depicted scene (Experiment 3). These findings provide novel evidence that kinematically-identical actions can elicit different predictions depending on the social context in which they occur.

## Introduction

Predicting others' actions enables us to interact with others smoothly and efficiently^1^. For instance, when receiving change from a cashier, perceiving where and when they will reach enables us to meet their hand with our own in real-time seamlessly. According to *predictive processing* accounts of action perception, we identify concurrent actions by rapidly forming and updating hypotheses about them as they unfold^2–5^. These predictions depend upon contextual cues, which include information about the observed actor's environment^6^, intentions^7,8^ and dispositions^9^.

Contextual cues might enable observers to refine their predictions for unfolding actions by ruling out those that would be impossible, or at least challenging, given actor and environment properties^2^. Although research has explored the impact of a diverse range of contextual cues, there is little work on the role of social context cues in action prediction. Observers are sensitive to how contexts can expand^10,11^ or restrict^12,13^ others' potential actions^14^. This study explores whether an actor's social context can shape an observer's action predictions. Specifically, we test whether observers interpret kinematically identical actions differently depending on whether they occur within a joint or solo action context.

## Prediction in action observation

Compelling evidence for predictive models of action observation comes from *predictive bias* paradigms in which participants view a sequence of frames depicting an effector in motion toward an object^8^. The viewed effector disappears mid-motion, and the participant responds by indicating the point on the screen where the hand disappeared. These judgement paradigms are based on *representational momentum*^15^, or the tendency for observers’ location judgements of moving objects to be biased toward future—or predicted—locations. Predictive bias paradigms use this phenomenon to study observers’ expectations about others’ actions. These paradigms assume that if action perception is biased toward anticipated actions, then our judgements of overt action properties, such as an effector's location, might also be biased so that they are consistent with predicted actions.

Consistent with predictive models of action perception, location judgement paradigms have shown a temporal bias in action observation. Specifically, in-motion effectors are judged closer to their target than they are. In their seminal study, Hudson et al.^8^ asked participants to view a sequence of rapidly presented frames that depicted an arm moving toward within-reach objects. Participants verbally instructed the viewed actor to take or leave the viewed objects based on their graspability or colour. The authors argued that these instructions would create strong action expectations for the viewed hand and would, therefore, bias overt location judgements toward the predicted destination of the hand. Finally, after stimulus offset, participants were presented with a probe that appeared either (1) in the same location, (2) displaced in the predicted direction (according to the verbal instruction), or (3) displaced in the unpredicted direction, relative to the offset position of the previously viewed hand. For both graspability and colour-cued action instructions, participants were more likely to detect that the probe was in a different location to offset when it was displaced in the unpredicted (vs predicted) location. In sum, participants were less likely to detect probe displacement when it was in the expected direction.

Aspects of the environment and how these relate to the actor can also guide action predictions. According to Bach et al.'s^2^ *affordance-matching hypothesis*, action possibilities, or affordances^16^ can inform action understanding. Predictions about ambiguous in-progress actions are made according to which actions are possible, given the properties of (1) the environment and (2) the observed actor. Evidence for this kind of predictive processing comes from Ambrosini et al.^17^, in which both adults and infants viewed videos of an actor reaching toward a small or large ball. The actor either reached with the hand posture required to grasp the ball (a pinch/precision grasp or an open-hand/power grasp for the small and large balls, respectively) or with a closed fist. Overall, results showed that both adults and infants gazed at the target object sooner when the actor's hand posture matched the target object, as opposed to being in a closed fist. That is, affordance-consistent hand posture facilitated action predictions, as measured by anticipatory gaze.

In addition to facilitating action predictions, affordance-action compatibility can disambiguate actions that are difficult to identify based on kinematic information alone. Participants in Ambrosini et al.^18^ viewed videos of a hand reaching laterally toward one of two closely placed objects, with one affording a power grasp and the other affording a precision grasp. Predictions about the reach target (as measured by anticipatory gaze) were more accurate when the actor's hand posture matched the grasp afforded of the target (vs a closed fist). Moreover, a recent study by McDonough et al.^6^ found that hand posture (power vs precision) shaped both explicit predictions and effector location judgements toward objects that matched the seen hand posture. In other words, affordance matching appears to guide action predictions while also biasing perception of overt action properties toward afforded actions.

## The present study

Many of our day-to-day actions occur in social contexts that modulate them. While having a co-actor might help us move objects^11^, walking alongside another can impede us while passing through a doorway too narrow for two people^14^. Since third-party observers are sensitive to how social contexts modulate others’ action possibilities^14^, these modulations may factor into action understanding just as affordances^6^. We sought to test this possibility in the present study. We asked whether kinematically-identical actions can be perceived differently according to the social context in which they occur. To this end, we showed participants videos of an actor walking toward an object alone or with a partner near the object. We expected the stationary actor, who appeared as if they were awaiting the walking actor’s help, would induce a prior for coordination. For this reason, we anticipated predictive bias consistent with joint action videos depicting two actors.

## Experiment 1

In Experiment 1, we showed participants videos in which an actor walked toward a piece of furniture in two conditions: *solo*, in which the actor was the only person depicted, and *joint*, in which another actor was standing by, and looking at, the piece of furniture. We chose this scenario as it might represent one example of a social context disambiguating an individual’s in-progress action. A table, for example, in an empty room does not offer a strong prior for what an individual walking in its direction might do (even if they would walk all the way to it). However, seeing another person seemingly waiting for the walking actor to help lift the object might induce a strong prior for joint lifting. We expected to find predictive bias effects consistent with these assumptions. Specifically, we expected participants to judge the walking actor as further along her trajectory in the joint vs solo context.

### Method

#### Participants and statistical power

Based on McDonough et al.^19^, who reported predictive bias effects upward of *d* = 0.53, we conducted a power analysis in G*Power 3 (Faul et al. 2007^20^). The analysis revealed that for a paired samples *t*-test (or an equivalent *F* test), with alpha = 0.05 and power = 0.80, we required a sample of 30 participants to test for a main effect of social context. For counterbalancing purposes, we recruited 32 (*M*_AGE_ = 27.0, 14 female, 17 male, 1 other, 29 right-handed, 3 left-handed) participants.

**Figure 1:**
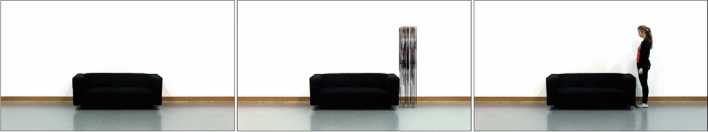
Initial frames from the same video stimulus across conditions, with the walking actor about to enter from the left. Solo videos in Experiment 1 showed the actor walking towards furniture only (left panel). In solo videos of subsequent experiments, participants saw a perceptually matched control in place of the actor (middle panel). Videos in the joint condition were unchanged across experiments and always showed an actor standing to the side while the other approached the object (right panel).

We collected data for the experiments reported in this study from Hungarian and Austrian samples. Participants received vouchers worth 1500 HUF (~ 4 EUR) for participation in Hungary or 10 EUR in Vienna. Participants provided informed consent before participating. We obtained ethics approval from the United Ethical Review Committee for Research in Psychology (EPKEB) in Hungary and the Psychological Research Ethics Board (PREBO) in Austria. We conducted all experiments in accordance with the Declaration of Helsinki (Fig. 1).

#### Stimuli

**Figure 2:**
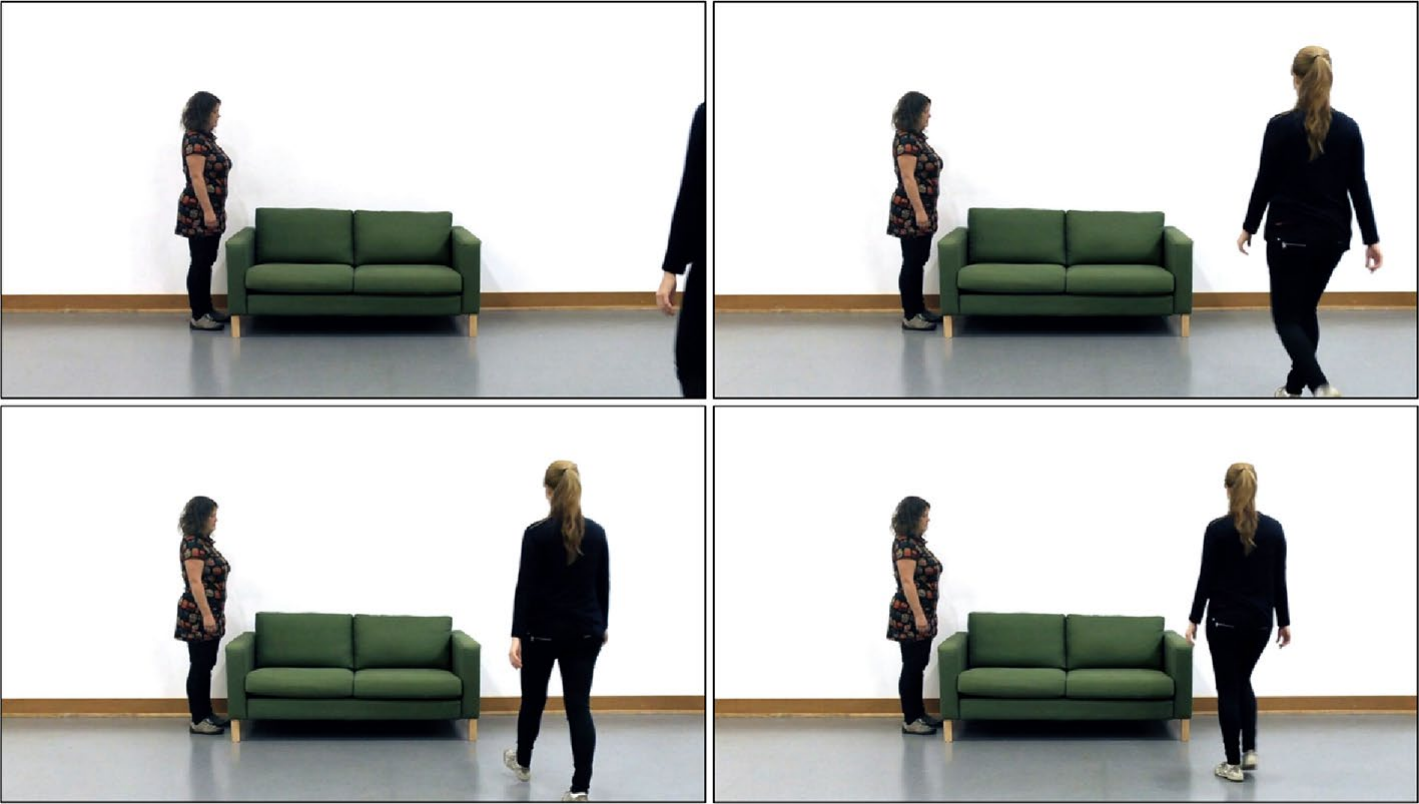
Frames 10, 35 (top), 45, and 55 (bottom) of a joint context video. The latter three frames were stopping points (45 and 55 in Experiment 1, 35 and 55 in all other experiments).

Stimuli were video clips depicting an actor walking toward one of four pieces of furniture (two couches, two tables) placed against the wall opposite the camera. We filmed the actors from the perspective of someone standing behind them as they walked away toward the objects. We filmed walks from each actor (with the other being stationary), but only from one side of the screen each (i.e., actor one always approached from the left of screen while actor two approached from the right). The actor either walked to the object's edge or slightly closer to its centre—a quarter of its width inward from the edge. All videos were filmed in the joint condition, with the second actor standing by the object. We made solo versions of each video by digitally removing the standing actor from the scene so that the action kinematics remained identical between social context conditions. In all, the combination of furniture (4), actor (2), walking target (edge/inner; 2), and social context (2) resulted in a total of 32 videos (Fig. 2).

To deal with the variance in walking speeds between clips, we re-timed each video so that the time from when the actor appeared on-screen to when they stopped walking was exactly 2 s (or 60 frames at 30 frames-per-second).

#### Procedure

Participants sat approximately 40 cm in front of a 21.5-in. 1080p touchscreen computer monitor. The experiment began with participants reading instructions that outlined the trial and response structure. Within these instructions was text contextualising the videos, which read: *“In this task, you will watch videos in which people will walk toward objects”*. Each trial began when participants pressed the C and 1 (number pad) keys (indicated with yellow markers) on a standard QWERTY keyboard with their left and right hands, respectively. This requirement stopped participants from tracking the actor’s location with their fingers. Participants needed to hold these keys until the end of the video; otherwise, it would terminate early, and participants would receive feedback reminding them to keep the keys held down until the end.

The videos always ended before the actor reached the end of her walk. Each video ended at either 45 or 55 frames (see Fig. 2 for stopping point examples). Following the video, a blank screen with only the text “LOCATION” prompted participants to make a location judgement on the next screen (for 100 ms). Next, participants saw a still frame from the preceding video. This frame was taken from the moment before the walking actor appeared on screen for that video. So, for the solo context videos, the frame featured an object only, while in the joint context, both the object and the second actor were present. This frame stayed on screen until the participant made a touch response indicating the location at which they last saw the actor before the video ended. We asked participants to touch the location between the actors’ shoulders to ensure somewhat consistent judgements between participants.

Next, participants saw a text prompt (“DESTINATION”) for 100 ms, after which they had to make a two-choice keypress response to indicate at which point on the x-axis they thought the actor would arrive. The two options were a quarter of the object’s width away from the edge that the actor approached. We showed these options on the same still frame as the location judgement but with red and blue markers at the two possible destinations. Participants indicated their judgement by pressing either the Z or 3 (number pad) keys on the keyboard, on which we placed red and blue stickers to correspond to the markers. We chose these two locations (despite the actor never actually walking to the point outside from the object’s edge) to elicit and capture explicit predictions from participants. We report these data in the Supplementary Materials.

Participants began by responding to four practice trials—two from each social context. Subsequently, participants responded to 256 experimental trials across 32 trial blocks. Each of the 32 videos appeared eight times, stopping at 45 and 55 frames four times each. We counterbalanced the side on which each actor walked throughout the videos by digitally flipping them for half of our sample.

Solo and joint trials were blocked so that participants saw all the videos for one social context before the other. We opted for this approach—rather than intermixing trials—to distinguish between the two contexts more clearly. Since people form predictions based on others’ past behaviour^9^, we thought it possible that participants' expectations of joint (or solo) actions could spill over into subsequent trials. The order of the social context was counterbalanced between participants.

### Results and discussion

We excluded data on the trial level when either the x- or y-coordinate for participants’ touch responses was more than 2.5 standard deviations from the global sample mean for each stimulus in each condition. We removed outliers by stimulus since we expected variation at this level due to the different kinematics each video depicted. However, since participants only saw each unique stimulus four times per experiment, we detected extreme values using means and standard deviations at the sample rather than participant level.

Additionally, we excluded participants for whom more than 20% of touch responses were invalid due to missing data or not meeting the standard deviation cutoff described above. Finally, we excluded any participants for whom we had zero valid responses in any condition of interest. In all, we retained 88% of the collected data. For included participants, we retained an average of above 95% (SDs < 1%) of trials per social context for this experiment as well as across all further experiments. We used the *afex*^21^ R package for the reported analyses.

We aggregated trial data for each participant at the stimulus level within each condition *before* aggregating data to the condition level. We did so because, otherwise, trial-level data exclusions could unbalance trials concerning object and actor and thus skew condition means. We coded coordinates so that y-values represented the distance of touches from the bottom of the screen, while x-values values indicated distance from the centre. Since actors approached from the bottom corner toward the centre of the screen, this coding meant that higher y values and lower x values indicated points further along the actor’s path. Although actors did move on the x-axis during their walks, we found that x-axis data were an unreliable measure of predictive bias across our experiments. We report x-axis analyses in the Supplementary Materials.

**Figure 3:**
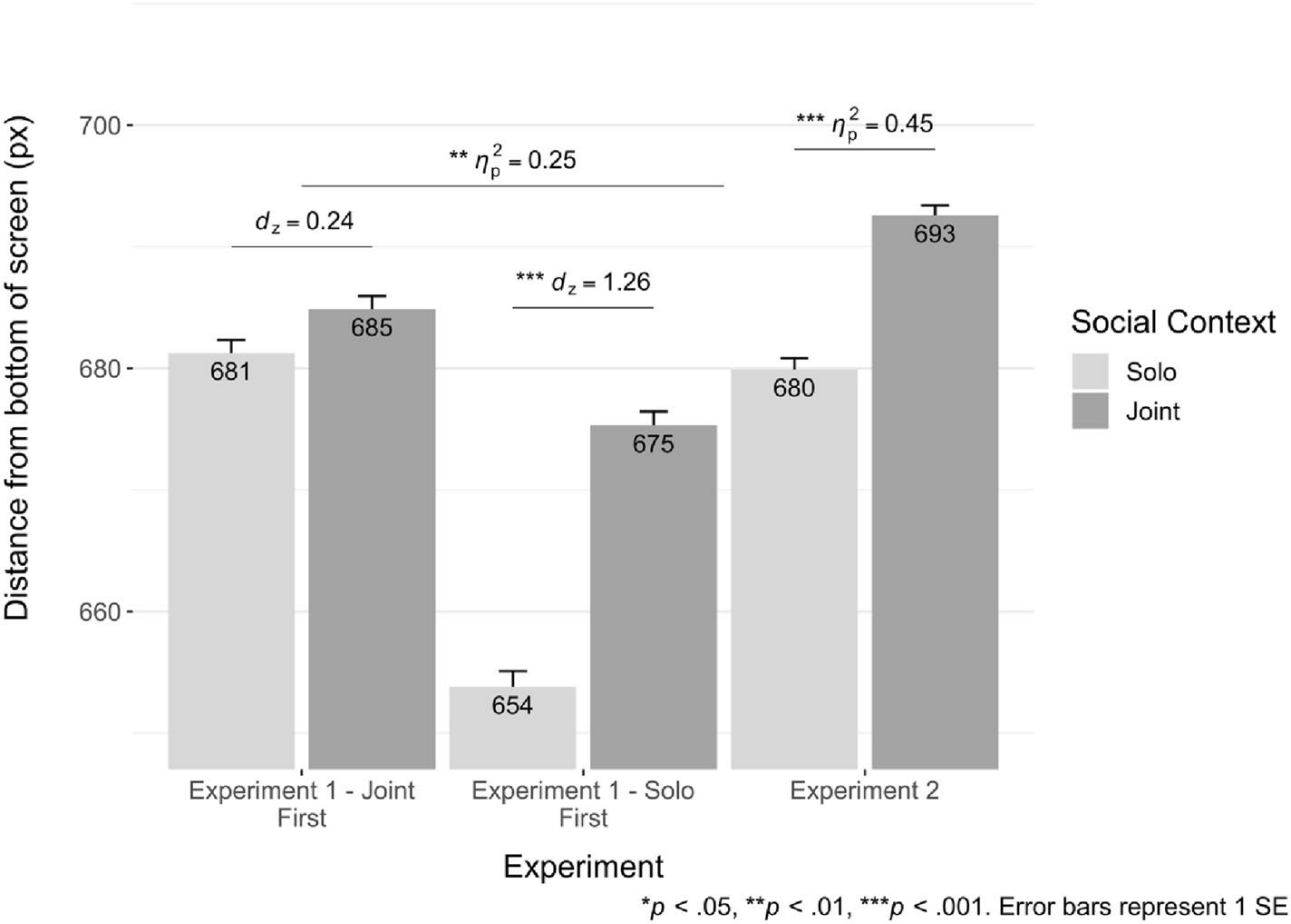
Social Context effect in Experiments 1 and 2. Labelled values are condition means.

To test whether social context influenced touch location judgements, we subjected y-coordinate data from touch responses to a (2)(Social Context: Joint v Solo)  × (2)(Frame: 45 vs 55) × (2)(Target: Inner vs Edge) × 2(Condition Order: Joint First vs Solo First) ANOVA. We found a significant main effect of Social Context *F*(1,27) = 17.5, *p* < 0.001, *ηp*^2^ = 0.39 in y-axis data (Fig. 3). Participants judged actors to be further along their path in joint vs solo videos despite the identical portrayed kinematics. Interestingly, this Social Context effect interacted with Condition Order, *F*(1,27) = 8.8, *p* < 0.01, *ηp*^2^ = 0.25. While the Social Context effect was significant for participants who watched solo videos first, *t*(14) = 4.89, *p* < 0.001, *dz* = 1.26, there was no effect for those who watched them following the joint videos, *t(*13) = 0.89, *p* = 0.39, *dz* = 0.24. This interaction likely indicates that seeing the joint videos first had some carry-over effect to subsequent solo videos. While responses to joint videos did not differ according to Block Order, *t*(27) = 0.78, *p* = 0.44, *dz* = 0.15, participants made higher (more predictive) touch responses to solo videos if they viewed them second *t*(27) = 2.08, *p* = 0.05, *dz* = 0.39.

We also found effects of Frame and Target (*p*s < 0.001), with participants making higher touches for inner-target and later-stopping videos. The Frame effect confirmed our assumption that higher touches would correspond with later points in the video. We do not offer an interpretation for the main effect of Target since the actors’ actual locations differed depending on the target they approached. All remaining effects were not significant (*p*s > 0.14).

In sum, Experiment 1 provided some support for our hypothesis that social contexts can guide action predictions. We found that videos of kinematically-identical walking actions elicited relative predictive bias when another actor was present vs. when they were not. Although the observed order effect aligns with other results showing that joint task representations can carry over to subsequent solo tasks [^22^; Exp 3], we interpret it with caution given that it was neither predicted nor the subject of our experiment. Instead, we sidestepped the context carry-over effect in subsequent experiments by showing all participants the solo videos first.

## Experiment 2

An alternative explanation for our social context effect in Experiment 1 is that the presence of a second actor introduces a low-level perceptual effect rather than a predictive cue to the walking actors’ actions. For example, the second actor might have drawn participants’ attention to a point higher on the screen and biased touch responses accordingly. We sought to rule out low-level explanations in Experiment 2 by replacing solo videos with ones in which a perceptually matched image appeared where the actor would be, rather than blank space. As in Experiment 1, we expected participants to judge actors to have been further along their trajectory in joint vs solo contexts.

### Method

#### Participants

To determine the sample size for Experiment 2, we conducted a power analysis using data from Experiment 1. We found that to detect a Social Context effect of ηp^2^ = 0.375 with power = 0.80, we would need a sample of 25 participants. This effect size is slightly smaller than the one reported in Experiment 1 because, when planning Experiment 2, a bug in the data exclusion code filtered out a small number of valid trials for Experiment 1, thus changing the effect size we initially computed. To account for potentially smaller effects due to removing perceptual feature differences between joint and solo stimuli, we recruited a larger sample of 36 participants (*M*_AGE_ = 27.1, 17 female, 19 male, 1 other; 28 right-handed, 7 left-handed,1 other). We preregistered this experiment at https://osf.io/knz8f. Due to an error in data collection, we recruited an extra participant whose data were excluded.

#### Stimuli

Stimuli were the same as in Experiment 1, except we edited the solo videos to include an unidentifiable but perceptually matched figure to the actor (Fig. 1). We did so using a similar method to Vestner^23^ by taking the original images of our static actors and randomly rearranging the pixels by column.

#### Procedure

The experiment was the same as in Experiment 1, with the following exceptions. First, since none of our factors predicted participants’ explicit destination judgements in Experiment 1 (Supplementary Materials), we removed these explicit judgments from this experiment. Second, we kept only the edge walking target videos since they better resembled a scenario in which actors would interact with the object. These changes reduced the total number of trials to 128. Third, we changed the earlier stopping point from frame 45 to frame 35 to increase the variability and reduce the visual similarity of the stopping points between videos. Finally, to avoid order effects as in Experiment 1, we showed all participants the solo videos first.

### Results and discussion

We employed the same data-cleaning procedure as in Experiment 1. This procedure excluded the data of two participants. These measures retained 92% of the collected data.

Critically, we replicated the focal social context effect, *F*(1,33) = 27.1, *p* < 0.001, *ηp*^2^ = 0.45 (Fig. 3). In other words, even when solo-context videos contained perceptually-matched non-social controls in place of actors, participants made higher, more predictive touches in joint-context videos. The frame effect, *p* < 0.001, did not interact with social context, *p* = 0.13.

## Experiment 3

Experiments 1 and 2 found that even when the task instructions only specified that the actors would walk toward objects, adding social context cues—in this case, a person waiting by a piece of furniture—elicited stronger action predictions. In Experiment 3, we explored whether the social context effect would remain even when the task instructions specified that the two actors would perform a solo action that was not lifting (placing a sticker on the sofa).

Previous work has found that contextual cues can override conflicting pre-cues to others’ actions in forming predictions. In Ambrosini et al.^24^, observers looked ahead to objects compatible with the reaching actor’s hand posture even when she gazed toward a different object. In Experiment 3, we sought to test whether observers base predictions on expected joint actions even after receiving conflicting information about the upcoming action. This time, the task preamble specified that actors would walk toward objects to ‘place a sticker on them’. Of interest was whether the initial cue to the action (sticker placement) would be overridden by the expectations participants might spontaneously generate in the joint condition.

### Method

#### Participants

We recruited an additional 36 participants for this experiment to ensure equal sample sizes for cross-experimental between-subjects comparisons (*M*_AGE_ = 26.7, 20 female, 15 male, 1 other; 29 right-handed, 7 left-handed). Sensitivity analyses revealed that with total sample sizes of at least 64, we could detect between-subjects effects upward of *ηp*^2^ = 0.11 with power = 0.80. Between-subjects analyses in Experiments 3–5 were not pre-registered.

#### Stimuli

Stimuli were the same as in Experiment 2.

#### Procedure

The procedure for Experiment 3 was identical to Experiment 2, except that the preamble now specified that the actors would lift the object with the following text: *“In this task, you will watch videos in which people will walk toward objects in order to place a sticker on them”*.

### Results and discussion

Data from three participants failed to meet the exclusion criteria outlined in Experiment 2. In all, we retained 88% of the data collected in Experiment 3.

**Figure 4:**
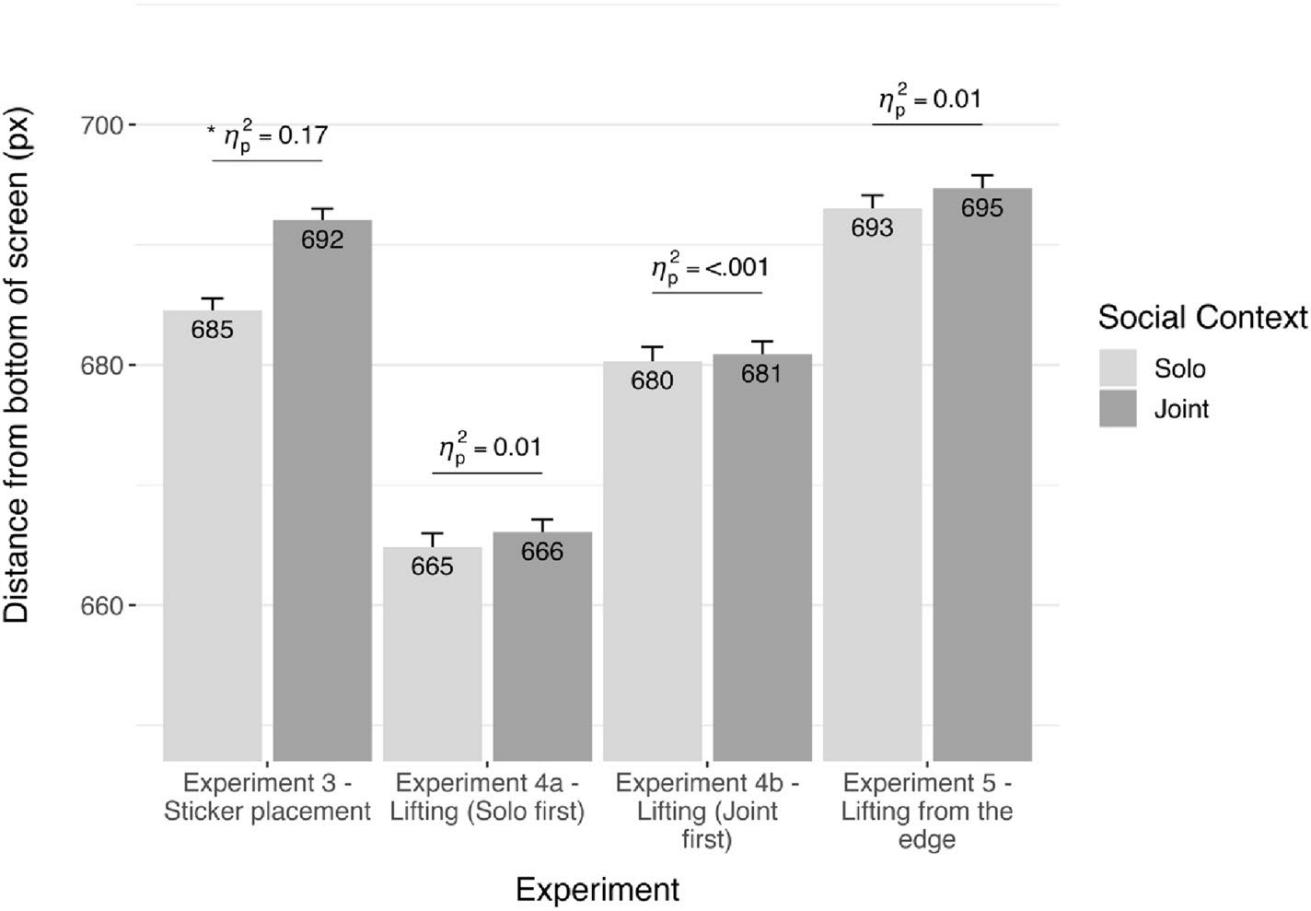
y-axis data for Experiments 3–5. Labelled values are condition means.

As in Experiment 2, we found that participants made higher touches in response to joint context videos, *F*(1,31) = 6.51, *p* = 0.02, *ηp*^2^ = 0.17 (Fig. 4). While the main effect of Frame was again significant, it did not interact with Social Context (*p* = 0.30).

To test whether the Social Context effect differed from Experiment 2, we subjected y-axis data to a (2)(Social Context: Joint v Solo) × 2(Experiment: 2; walking instruction v 3; sticker instruction) cross-experiment mixed ANOVA. We did not find a main effect of Experiment, *p* = 0.79. However, the main effect of Social Context was significant, *F*(1,65) = 27.7, *p* < 0.001, *ηp*^2^ = 0.30. We did not find two-way interaction between Experiment and Social Context (*p* = 0.21).

In sum, data from Experiment 3 suggests that expectations concerning joint actions based on perceptual information can guide action prediction even when an earlier cue specifies a different, solo, action.

## Experiments 4a and 4b

Finding that the social context effect remained despite a conflicting action specified in the instructions raises a question as to whether our effect is indeed driven by action prediction or whether participants simply had a general bias in responding closer to the other actor. Such effects occur in perceptual binding paradigms^25^, in which individuals judge two stationary individuals to be closer together than they are when they make eye contact compared to when they do not. Critically, perceptual binding effects do not require individuals to anticipate a specific action. In this experiment, we sought to test whether perceptual binding-like effects could explain the social context effect in our study.

In Experiment 3, we proposed that the social context effect remained because, although we specified sticker placement, the videos more strongly resembled a joint lifting context than a solo sticker placement context. In Experiments 4a and 4b, we tested whether specifying that actors would lift the object would moderate the social context effect. Since the specified lifting action would, this time, match the observed scenario equally, we might expect predictive bias to be equal in both conditions. If, however, the effects observed in Experiments 1–3 were driven by perceptual binding, we should still find a difference between the two conditions, with the actor in the joint condition judged as closer towards the other.

Below, we report the results of two experiments—one in which participants saw solo videos first as in Experiments 2 and 3 (Experiment 4a), and one in which they saw them second (Experiment 4b). In both, participants read in the instructions that the actors were walking toward the objects to lift them. We added the joint-first experiment after the solo-first data produced results suggesting that our manipulation may have induced carry-over effects. For simplicity, we report data from these experiments together.

### Method

#### Participants

We aimed for a sample of 36 participants (*M*_AGE_ = 27.2, 23 female, 13 male, 32 right-handed, 4 left-handed) for Experiment 4a (solo first) to facilitate cross-experimental analysis with Experiment 2. We collected another 36-participant sample for Experiment 4b (*M*_AGE_ = 26.2, 20 female, 15 male, 1 other, 28 right-handed, 8 left-handed).

#### Stimuli

Stimuli were the same as those in Experiments 2 and 3.

#### Procedure

The procedure for Experiments 4a and 4b were identical to Experiments 2 and 3, except that the preamble now specified that the actors would lift the object with the following text: *“In this task, you will watch videos in which people will walk toward objects in order to lift them”*. In Experiment 4a, participants saw solo videos first while in Experiment 4b they saw joint videos first.

### Results and discussion

Applying exclusion criteria from the previous experiments, we excluded data from three participants in Experiments 4a, retaining 90% of the collected data. In Experiment 4b, we retained 85% of the collected data after excluding four participants and losing an additional participant’s data due to a technical error.

Analysis of y-axis data from participants who completed the solo block first did not reveal a Social Context effect, *F*(1,32) = 0.3, *p* = 0.59, *ηp*^2^ = 0.01. Cross-experimental analysis with Experiment 2 data revealed an Experiment × Social Context interaction showing that the Social Context effect was significantly larger in Experiment 2, F(1,65) = 7.96, p < 0.01, ηp^2^ = 0.11, achieved power = 0.79. Surprisingly, we found a main effect of Experiment (*F*(1,65) = 7.09, *p* = 0.01, *ηp*2 = 0.10, achieved power = 0.76), showing that y-axis touches were lower or less predictive in Experiment 4a across Social Context conditions.

One explanation for this overall suppression in 4a is that participants perceived lifting to be impossible in the solo context and thus made less predictive touches. This suppression might have carried over to the subsequent joint condition. Data from Experiment 4b, in which participants saw the joint videos first, support this explanation. Again, we did not find a Social Context effect in 4b, *F*(1,30) = 0.01, *p* = 0.91, *ηp*^2^ < 0.001. Although the comparison between the solo context data from Experiment 4a and joint context data from 4b did not yield a between-subjects main effect of Social Context, *F*(1,61) = 3.12, *p* = 0.08, *ηp*^2^ = 0.05, achieved power = 0.73—participants who viewed joint context videos (i.e., those in 4b) made numerically-higher touches on the y-axis (Fig. 4).

Although not predicted, the large difference in anticipation between Experiments 2 and 4b is of theoretical interest. It is in line with the results of other studies showing that prediction for unfolding actions is suppressed when the action appears impossible [e.g. because the actor is reaching toward an object with an incompatible hand posture^18^]. Importantly, the pre-task instructions modulated participants’ responses differently across social contexts in Experiments 2 and 4, implying that the social context effect depends on action expectations, not merely on perceptual binding effects.

## Experiment 5

In Experiment 5, we attempted to test whether we could elicit action prediction in both social contexts (thus suppressing the social context effect) by specifying a lifting action plausible in both social contexts. This time, we instructed participants that they would watch videos in which actors would lift objects from the edge nearest to themselves. We expected participants to interpret this as the actor only raising one side of the furniture while the other remains grounded. For joint videos, we were agnostic as to the kind of lifting participants would expect—either solo as described above, or joint. In either case, we expected a suppression in the social context effect due to introducing an action prediction in the solo condition.

### Method

#### Participants

We recruited a new sample of 36 participants (*M*_AGE_ = 28.9, 17 female, 9 male, 1 other; 32 right-handed, 4 left-handed) for Experiment 5. Again, we chose this sample size to facilitate cross-experimental analyses with Experiments 2 and 4a.

#### Stimuli

Stimuli were the same as in Experiments 2–4b.

#### Procedure

The procedure for Experiment 5 was identical to Experiments 2–4b, except that the preamble now specified that the actors would lift the object with the following text: *“In this task, you will watch videos in which people will walk toward objects in order to lift them from the edge closest to themselves”*.

### Results and discussion

Data cleaning measures retained 92% of the data we collected in Experiment 5, with two participants being removed from analyses.

We did not find a main effect for Social Context in Experiment 5, *p* = 0.50 (Fig. 4). A cross-experiment ANOVA with y-axis data from Experiment 2 did not reveal a main effect of Experiment (p = 0.66). However, there was a main effect of Social Context driven by Experiment 2 data, *F*(1,66) = 18.3, *p* < 0.001, *ηp*^2^ = 0.22. The Social Context x Experiment interaction was significant—with the Social Context effect being significantly smaller in Experiment 5, *F*(1,66) = 11.1, *p* < 0.01, *ηp*^2^ = 0.14, achieved power = 0.89.

To rule out that the suppression of the Social Context effect resulted from a carry-over effect like in Experiment 4a, we tested whether y-axis data from the first (solo condition) halves of the task differed between Experiments 2 and 5. This test did not reveal any evidence for a suppression effect in the solo blocks that could have carried over, *F*(1,66) = 2.41, *p* = 0.13, *ηp*^2^ = 0.04. Rather, responses in the solo context in Experiment 5 were numerically higher. Additionally, we found a cross-experimental difference with Experiment 4a—participants in Experiment 5 made higher touches on the y-axis overall (*F*(1,65) = 9.32, *p* < 0.01, η*p*^2^ = 0.13, achieved power = 0.87), but this did not differ across social conditions, *p* = 0.39.

Experiment 5 shows that the predictive bias difference between social contexts comes from an expected action rather than perceptual-binding-like effects. Specifically, we found that although the depicted scenario lends itself to predictive bias in joint contexts, explicitly instructing participants that actors would perform an action that is plausible in both solo and joint conditions (c.f. Experiment 4a) and is also visibly consistent with the video (c.f. Experiment 3) produces predictive bias in either social context.

## General discussion

While a growing body of evidence shows that different types of cues can guide the prediction of others' actions, little is known about whether others' social contexts serve a similar role during action observation. In this study, we asked participants to indicate the last-seen location of actors walking toward pieces of furniture alone or with another person standing by. In line with our predictions, participants judged actors to be further along their trajectory when the scene featured another actor such that it was plausible to assume the two actors would lift the object they were walking towards (Experiments 1 and 2).

Taken together, our y-axis data indicate that observers can disambiguate kinematically identical actions using social context cues. Results from Experiments 1 and 2 show that a co-actor inviting a particular joint action—in this case, joint lifting—can elicit a strong prior for predicted joint action. In Experiment 3, we found that the co-actor elicited predictive bias for joint action even when we specified that actors would perform a different action (sticker placement) at the end of their trajectory.

Critically, Experiments 4a–5 indicate that the social context effect is driven by action expectations elicited by seeing a co-actor. Experiment 5, in which we specified an action possible in both social conditions (lifting an object from the edge) suppressed the social-context effect by producing equally predictive responses in either condition. However, when we specified an action that would have been difficult-to-impossible in a solo context (lifting the object; Experiments 4a and 4b), we found touches to be less predictive overall compared to when the action was unspecified (Experiment 2). Experiment 4b showed that this overall suppression in 4a came from the solo context and carried over to the joint, since it was no longer present when participants saw the joint videos first.

Interestingly, we found a social context effect despite the actors having solo actions, such as sitting, available to them. This is likely due to the joint scene having a clearly associated action based on how the stationary actor stood—in this case, joint lifting. Additionally, observers might interpret a waiting co-actor as a powerful cue for helping or coordinative actions. Conversely, the solo videos did not have an equally strong cue for what the walking actors would do at the end of their trajectory. This was true even when we instructed participants that the actors would place a sticker on the object. One explanation for why the social context effect remained in Experiment 3 is that how and where actors would place the sticker is unclear in the video stimuli. In contrast, the presence of a co-actor together with an object might elicit more specific priors for the actor’s subsequent movements. Experiment 5 supports this explanation—when we specified an action along with a clear course of action in both contexts, responses were no more predictive in the joint vs solo context.

While data on the y-axis provided clear and interpretable evidence for predictive bias, x-axis data did not produce converging effects (Supplementary Materials). The apparent absence of x-axis effects might have been due to the actors deviating relatively little laterally—both in their surroundings and on-screen. Further, actors reached the edge of the objects quite quickly on the x-axis, which might have created a ceiling for predictive bias on this dimension.

Our work builds on a growing literature showing the predictive nature of action understanding. Perceivers integrate multiple cues, such as kinematics, others’ surroundings^6^, and explicitly provided intentions^7^ to form constantly-updated expectations about others’ actions. These expectations, in turn, bias our perception of low-level action kinematics toward the predicted outcome. Here, we have shown that people also integrate information about potential action partners, forming priors for coordination when the situation invites it.

One question our study raises is how people resolve conflicts between verbal and perceptual information about actions in different contexts. Whereas participants in Experiment 3 seemed to anticipate joint action despite being instructed that actors would place a sticker on the object, the preambles concerning lifting (Experiments 4–5) suppressed the social context effect. A potential explanation is that cues are weighted according to the context in which they appear. For example, an observer might rely on cues regarding kitchen utensils (e.g., whether someone has a knife) when watching someone cook but not when doing their laundry. Similarly, the sticker placement cue in Experiment 3 might have had less relevance than lifting cues that were ostensibly more related to the stimulus context in our study.

Another open question is whether having visibly disinterested or impaired^26^ co-actors would suppress our social context effect. Future research could also investigate whether there are situations in which the addition of another person obfuscates a likely solo action and thus produces the reverse effect. For instance, an observer might expect someone to take a particular object unless another person, who looked like the object belonged to them^12^, was present.

In sum, our study builds on existing action prediction literature^6,8^ by showing that social context is a powerful cue to understanding others’ actions. Specifically, we show that people perceive kinematically identical actions differently according to others’ social contexts, which provide insight into their goals and intentions.

### Supplementary Information

Supplementary Information.

## Data Availability

Data are available at https://osf.io/7x3sw/.
