# Supplementary material for: Predicting others’ actions from their social contexts
Source: Sci Rep. 2023 Dec 12;13:22047. doi: 10.1038/s41598-023-49081-6 (PMC10716130; doi:10.1038/s41598-023-49081-6)
Supplement: Supplementary file 1 — Supplementary Information. [file 41598_2023_49081_MOESM1_ESM.pdf]

Supplementary Materials

## Explicit Destination Judgements in Experiment 1

To test whether Social Context also affected participants' explicit judgements of actors' destinations, we fit a mixed-effects logistic regression on participants' responses. Since it was not possible for the model to converge with all our Experiment 1 factors, we tested only for the effect of Social Context and its interaction with Condition Order, as these factors predicted responses on the y-axis. Neither Social Context. Condition Order, nor their interaction predicted participants' explicit destination judgements (zs <1.5, *p*s > .14). This is unsurprising considering that the two response options were between locations at or away from the objects (Figure 1) – participants chose the location at the object most (74%) of the time.

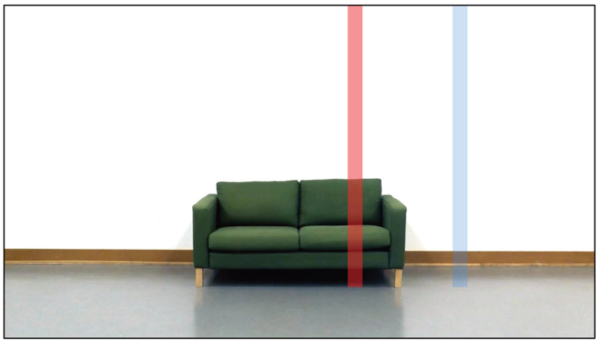

Figure 1. Destination Judgement Probe in Experiment 1

**x-axis data**

Touches on the x-axis were did not reliably indicate predictive bias across our experiments (Figure 2). Although we did find that participants touched closer to the centre-of-screen in Experiment 1, $F(1,27) = 4.33$, $p = .047$, $\eta p^2 = 0.14$, we did not replicate this effect in Experiment 2 or 4a (*p*s > .09) . Furthermore, while Experiments 3, and 5 also produced significant Social Context effects on the x-axis (*ps* <.05), the x-axis effect in Experiment 4b was reversed, indicating that it was, at least in part, driven by practice or time-on-task effects, $F(1,30) = 4.08$, $p = .053$, $\eta p^2 = 0.12$.

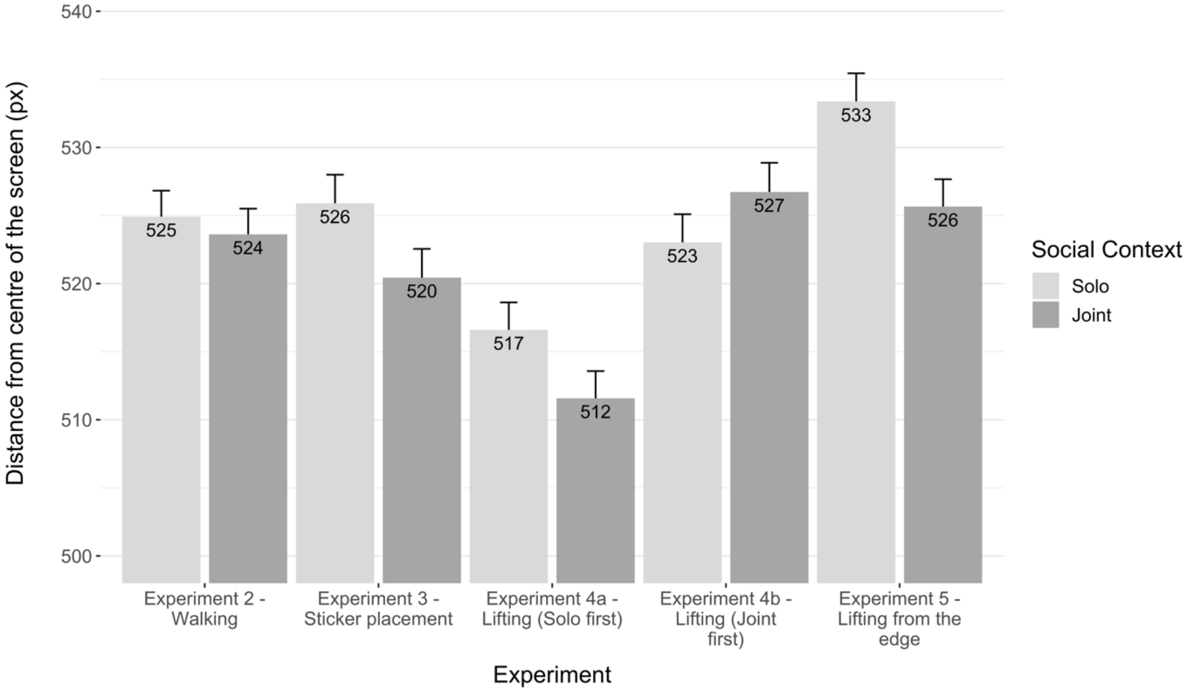


Figure 2. x-axis Data across Experiments 2-5
